# Opportunistic Diagnostics of Dental Implants in Routine Clinical Photon-Counting CT Acquisitions

**DOI:** 10.3390/jimaging11070215

**Published:** 2025-06-30

**Authors:** Maurice Ruetters, Holger Gehrig, Christian Mertens, Sinan Sen, Ti-Sun Kim, Heinz-Peter Schlemmer, Christian H. Ziener, Stefan Schoenberg, Matthias Froelich, Marc Kachelrieß, Stefan Sawall

**Affiliations:** 1 Department of Conservative Dentistry, Clinic for Oral, Dental and Maxillofacial Diseases, University Hospital Heidelberg, Heidelberg University, Im Neuenheimer Feld 400, 69120 Heidelberg, Germany; 2 Department of Oral- and Maxillofacial Surgery, Clinic for Oral, Dental and Maxillofacial Diseases, University Hospital Heidelberg, Heidelberg University, Im Neuenheimer Feld 400, 69120 Heidelberg, Germany; 3 Department of Orthodontics, University Hospital of Schleswig-Holstein, Arnold-Heller-Straße 3, 24105 Kiel, Germany; 4 Department of Radiology, German Cancer Research Center (DKFZ), Im Neuenheimer Feld 280, 69120 Heidelberg, Germany; 5 Department of Clinical Radiology and Nuclear Medicine, University Hospital Mannheim, Theodor-Kurz-Ufer 1-3, 68167 Mannheim, Germany; 6 Medical Faculty, Heidelberg University, Im Neuenheimer Feld 672, 69120 Heidelberg, Germany; 7 Division of X-Ray Imaging and CT, German Cancer Research Center (DKFZ), Im Neuenheimer Feld 280, 69120 Heidelberg, Germany

**Keywords:** radiography, dental, tomography, X-ray computed, dental implants, incidental findings

## Abstract

Two-dimensional imaging is still commonly used in dentistry, but does not provide the three-dimensional information often required for the accurate assessment of dental structures. Photon-counting computed tomography (PCCT), a new three-dimensional modality mainly used in general medicine, has shown promising potential for dental applications. With growing digitalization and cross-disciplinary integration, using PCCT data from other medical fields is becoming increasingly relevant. Conventional CT scans, such as those of the cervical spine, have so far lacked the resolution to reliably evaluate dental structures or implants. This study evaluates the diagnostic utility of PCCT for visualizing peri-implant structures in routine clinical photon-counting CT acquisitions and assesses the influence of metal artifact reduction (MAR) algorithms on image quality. Ten dental implants were retrospectively included in this IRB-approved study. Standard PCCT scans were reconstructed at multiple keV levels with and without MAR. Quantitative image analysis was performed with respect to contrast and image noise. Qualitative evaluation of peri-implant tissues, implant shoulder, and apex was performed independently by two experienced dental professionals using a five-point Likert scale. Inter-reader agreement was measured using intraclass correlation coefficients (ICCs). PCCT enabled high-resolution imaging of all peri-implant regions with excellent inter-reader agreement (ICC > 0.75 for all structures). Non-MAR reconstructions consistently outperformed MAR reconstructions across all evaluated regions. MAR led to reduced clarity, particularly in immediate peri-implant areas, without significant benefit from energy level adjustments. All imaging protocols were deemed diagnostically acceptable. This is the first in vivo study demonstrating the feasibility of opportunistic dental diagnostics using PCCT in a clinical setting. While MAR reduces peripheral artifacts, it adversely affects image clarity near implants. PCCT offers excellent image quality for peri-implant assessments and enables incidental detection of dental pathologies without additional radiation exposure. PCCT opens new possibilities for opportunistic, three-dimensional dental diagnostics during non-dental CT scans, potentially enabling earlier detection of clinically significant pathologies.

## 1. Introduction

Dental imaging accounts for a significant proportion of all radiographic examinations in medicine. However, its contribution to the overall radiation exposure remains relatively low [1]. This is primarily due to the widespread use of two-dimensional imaging modalities in dental diagnostics, such as intraoral radiographs, panoramic radiography (OPT), and bitewing radiographs. These imaging techniques are routinely used in dental practice and already provide valuable information regarding potential pathologies. For example, bitewing radiographs can be used to detect caries located in the approximal spaces between teeth. Two-dimensional imaging modalities can also support implant planning to a certain extent. However, a common limitation of all these methods is the lack of a third dimension. They are summation images, meaning that anatomical structures are projected onto a single plane and superimposed thereon. As a result, depending on the angle of acquisition, certain pathological findings, such as apical lesions at the root tips, may be obscured by superimposed structures and thus go undetected. Three-dimensional imaging techniques can overcome these limitations by providing volumetric data and enabling more accurate visualization of complex anatomical relationships. However, three-dimensional imaging techniques, such as cone beam computed tomography (CBCT), are only used in exceptional cases with strict indications due to their considerably higher radiation dose for patients. Conventional CT scanners, which were used for three-dimensional imaging before the advent of CBCT systems, offer limited spatial resolution, making it difficult to address many clinically relevant questions in dentistry [2]. Alternative imaging modalities, such as micro-computed tomography (micro-CT) systems, provide markedly higher spatial resolution but require substantially higher radiation dose levels and are typically limited to a field of view of only a few centimeters, making them impractical for clinical patient use.

A promising imaging modality to overcome the mentioned limitations is photon-counting computed tomography (PCCT), which represents a significant advancement in CT technology. Compared to traditional energy-integrating (EI) systems, PCCT offers substantial benefits in terms of spatial resolution, soft-tissue contrast, and radiation dose efficiency [3]. In conventional EI-based systems, incoming X-ray photons are absorbed by a scintillator, typically gadolinium oxysulfide (Gd_2_O_2_S), which subsequently emits optical photons that are directed to photodiodes via reflective lamellae, where the final signal is eventually generated. In contrast, photon-counting CT systems use direct detection within a semiconductor material, most commonly cadmium telluride (CdTe) [4,5]. Upon X-ray photon absorption, a charge cloud is generated and transported to electrodes or individual pixels via an applied bias voltage. High-speed electronics enable the detection of individual photons and their energy levels. Typically, the detected photons are classified into two to four discrete energy bins, allowing for dual- or even multi-energy acquisitions. Due to the high X-ray flux rates in clinical CT, which can reach up to 10^6^ photons/s/mm^2^, the electrodes used in photon-counting detectors are significantly smaller than those in conventional EI-based systems [4,5]. This design allows for either enhanced spatial resolution or a reduction in radiation dose. Early studies have demonstrated that PCCT provides exceptional image quality for dental structures, pathologic conditions, and bony structures of the periodontium [6,7]. An initial study using an experimental whole-body photon-counting CT illustrated the potential of this novel imaging technology for visualizing relevant dental structures [6]. Another study, conducted using the first clinically approved photon-counting CT, confirmed its suitability for complex endodontic diagnostics, demonstrating that even small endodontic structures can be visualized in high detail [8]. Additionally, research has shown that PCCT enables a significant reduction in radiation dose while simultaneously improving image quality compared to conventional CBCT using a dedicated dental protocol [7]. In this study, a PCCT with 1 mGy and a CBCT with 4 mGy were used. The results showed an overall significantly better assessment of dental structures with PCCT. Additionally, the CNRD was increased by up to 37% in PCCT [7]. Given these promising results, using opportunistic data, most likely acquired at a higher radiation dose compared to dedicated dental CBCT acquisitions, promises an even further increase in image quality. A recently published study investigated the suitability of PCCT for planning dental implants. Once again, PCCT demonstrated high precision [9]. However, all these studies have so far been conducted in vitro on pigs. Two major applications for this technology can be envisioned. Firstly, with increasing availability and accessibility, PCCT could potentially become the primary imaging modality for dental diagnostics in the future. However, this would likely require scanners that match cone beam CT (CBCT) systems in terms of usability and cost. This is particularly important because, in dental procedures, imaging is often needed directly during the course of treatment. Furthermore, if PCCT were to replace CBCT, and potentially also other two-dimensional imaging modalities, the increased number of examinations would likely exceed the capacity of PCCT scanners available in outpatient radiology practices. Secondly, for the first time, PCCT offers the prospect of opportunistic dental imaging. This means that PCCT scans performed for other clinical indications, where the teeth and surrounding structures are incidentally included, could also be utilized for dental assessment. This approach could facilitate the early detection of undiagnosed chronic pathologies, such as periapical periodontitis, leading to timely referrals to dental specialists. Early referral to dental professionals can have a positive impact on the quality of life of affected patients by enabling timely intervention and helping to prevent secondary complications. For example, the early treatment of chronic apical periodontitis can prevent the development of an abscess. In addition, early intervention may also have a favorable effect on the economic burden of dental diseases by reducing productivity losses, thus benefiting society as a whole [10]. Moreover, due to its superior soft-tissue differentiation, PCCT may enable the first truly artifact-free, three-dimensional visualization of peri-implant tissues. Therefore, the present study investigates for the first time the imaging quality of peri-implant tissues using clinically available in vivo data. Our hypothesis is that PCCT provides high image quality for peri-implant structures in vivo. Furthermore, we hypothesize that the application of metal artifact reduction (MAR) algorithms negatively impacts the immediate peri-implant region by reducing image clarity and differentiation. The hypotheses will be tested using opportunistic data obtained from scan protocols optimized for cervical spine imaging. These datasets will be reconstructed both with and without dedicated metal artifact reduction, and at multiple monochromatic energy levels known to influence artifact severity, followed by comprehensive quantitative and qualitative analyses.

## 2. Materials and Methods

### 2.1. Patient Population

Four patients who underwent a standard PCCT scan of the cervical spine at the University Medical Center Mannheim between May 2023 and May 2024 were retrospectively included in the study. In total, the patients had 10 dental implants, 3 in the maxilla and 7 in the mandible. The study was conducted in accordance with the Declaration of Helsinki and approved by the Ethics Committee II of the University of Heidelberg (ID 2021-659). Informed written consent was obtained from all subjects involved in this retrospective study after receiving both oral and written information regarding the use of their data for research purposes. Participation in this study was voluntary.

### 2.2. Image Acquisition and Reconstruction

A summary of all scan and reconstruction parameters is provided in Table 1. Initial image acquisitions were performed using the stock protocol for cervical spine imaging available at the scanner (Naeotom Alpha, Siemens Healthineers, Forchheim, Germany). All acquisitions were performed using a spiral trajectory and using the standard detector mode providing a detector pixel size in the center of rotation of about 300 µm, using a tube voltage of 120 kV and a dose of 13.2 ± 2.3 mGy (CTDI_32cm_) on average over all patients. Note that the diagnostic reference level for CT examinations of the cervical spine is 15.0 mGy in Germany [11].

Image reconstruction was performed using Quantum Iterative Reconstruction (QIR), the default iterative reconstruction of the scanner, at strength 3 and using the Qr64 reconstruction kernel (MTF_10%_ = 15.7 lp/cm). All data were reconstructed to a matrix of 1024 × 1024 voxels covering a field of view (FOV) of 160 mm, resulting in an axial voxel size of 156 µm and using a slice thickness of 0.4 mm and a slice increment of 0.2 mm. The FOV was placed to enclose the complete upper and lower jaw in all reconstructions.

A total of eight different reconstructions were performed per scan. In particular, virtual monochromatic images were reconstructed at energies of 70 keV, 100 keV, 150 keV, and 190 keV, respectively. Furthermore, all data were reconstructed with and without dedicated metal artifact reduction, termed iterative metal artifact reduction (iMAR), at the scanner [12]. Hence, 4 monochromatic energies were reconstructed with and without dedicated metal artifact reduction, amounting to a total of 8 reconstructions per patient.

### 2.3. Qualitative and Quantitative Image Analysis

**Figure 1:**
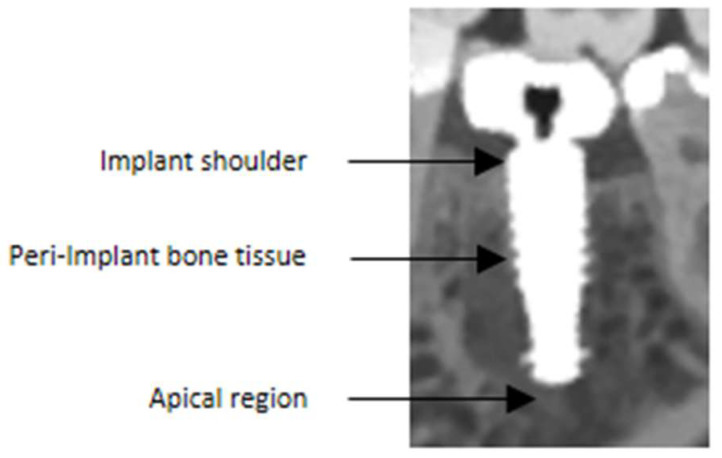
The assessment focused on specific anatomical regions of interest. Firstly, the region at the implant shoulder was evaluated, as it is critical for identifying the exact course of the bone and detecting potential peri-implant bone lesions. In addition, the peri-implant bone tissue and the implant-to-bone interface were analyzed, both of which are essential for detecting possible peri-implant pathologies. Lastly, the apical region was assessed, since persistent inflammation can occur in this area—particularly in cases of immediate implant placement—if extraction sockets are not adequately curetted. Note that this figure was magnified for illustrative purposes.

Qualitative image quality was independently assessed by two dentists (M.R., H.G.), each with more than 10 years of experience in reporting CBCT scans and 5 years in reporting PCCT scans. The examiners analyzed the entire stack of slices for each patient using a conventional DICOM viewer (Radiant DICOM Viewer 2023.1, Medixant, Poznań, Poland). Windowing and leveling were allowed. All evaluations were performed on 7the same monitor (RadiForce MX315W, 31.1 inch, EIZO, Mönchengladbach, Germany) in the same dark room. Examiners were blinded to each other. Image quality with respect to overall image quality as well as assessment of peri-implant bone tissue, implant shoulder, apex of the implant, and the overall image quality was evaluated using a five-point quality scale (1  =  excellent, 2  =  good, 3  =  moderate, 4  =  poor, 5  =  not visible) (Figure 1) [7]. Each examiner evaluated all reconstructions and implants. In total, each examiner completed 320 assessments. Quantitatively, voxel-wise differences across all evaluated monochromatic energies were determined, along with the mean CT values and standard deviations within cortical bone in a 5 mm elliptical region of interest near the implant.

### 2.4. Statistical Evaluation

Statistical analysis was performed using the R software package (version 4.3.1., R Foundation of Statistical Computing, Vienna, Austria). Continuous variables are expressed as mean ± standard deviation. Furthermore, inter-reader reproducibility was assessed using the intraclass correlation coefficient (ICC). The ICC is considered poor for ICC < 0.40, moderate to good for 0.40 < ICC < 0.75, and excellent for ICC > 0.75.

## 3. Results

### 3.1. Image Quality Quantification and Metal Artifact Reduction

**Figure 2:**
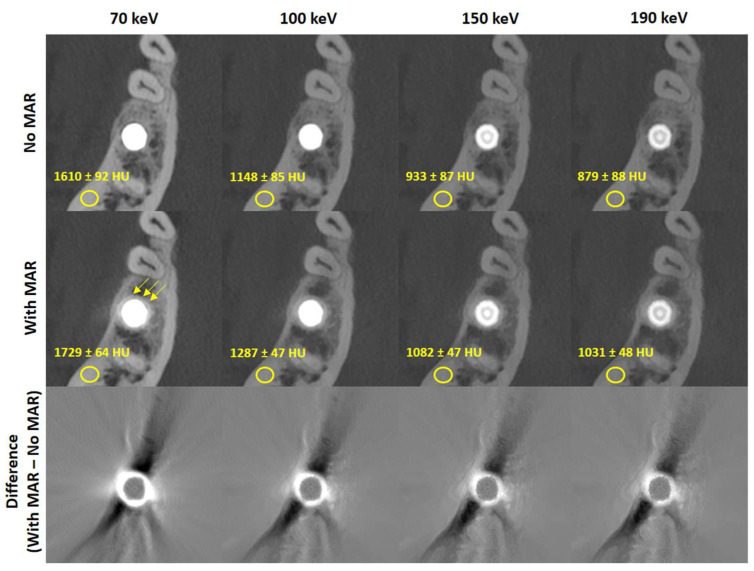
Exemplary reconstructions of one implant without (**top row**) and with (**middle row**) metal artifact reduction for all considered monochromatic energies as well as the voxel-wise difference among them (**bottom row**). The application of MAR seems to introduce a hyperdense quasi-homogeneous ring around the metal that is not present in the uncorrected reconstructions (arrows middle row) Reconstructions are windowed C = 3500 HU, W = 7000 HU, difference images are windowed C = 0 HU, W = 1800 HU.

Figure 2 presents axial CT slices of a representative implant reconstructed without MAR (top row), with dedicated MAR (middle row), and their voxel-wise difference (bottom row) across all evaluated monochromatic energies. It also reports the mean CT values and standard deviation measured in cortical bone within a 5 mm elliptically shaped region of interest in the vicinity of the implant. As expected, the mean CT values decrease with increasing monochromatic energy in both sets of reconstructions. However, the MAR images exhibit higher cortical bone CT values, potentially reflecting beam-hardening correction. This should be taken into account when estimating bone density for a second implant placed near an existing one. Similarly, the standard deviation in the ROI is lower in MAR reconstructions due to removed streak artifacts, as visible in the difference images. Notably, the implant itself shows negligible change, consistent with algorithms that segment and reinsert the original metal into the MAR-corrected volume [13,14]. However, the application of MAR seems to introduce a hyperdense quasi-homogeneous ring around the metal that is not present in the uncorrected reconstructions (arrows Figure 2). The aforementioned findings hold across all implants and patients, with the cortical mean CT values increasing by 12 % and the noise levels decreasing by 42 % on average in the MAR-corrected images.

### 3.2. Qualitative Analysis

**Figure 3:**
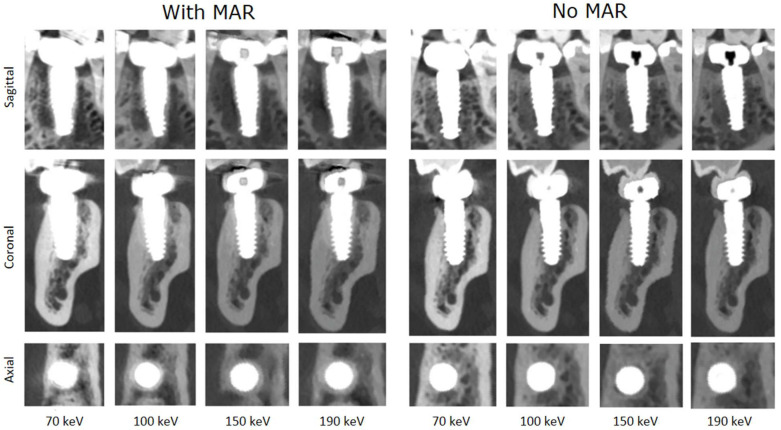
Systematic visualization of the different reconstruction protocols in sagittal, transverse, and axial planes of an implant in region 036. This standardized multi-planar approach allows for a consistent comparison of image quality and artifact expression across protocols and anatomical orientations (*C* = 3100 HU, *W* = 8500 HU).

**Figure 4:**
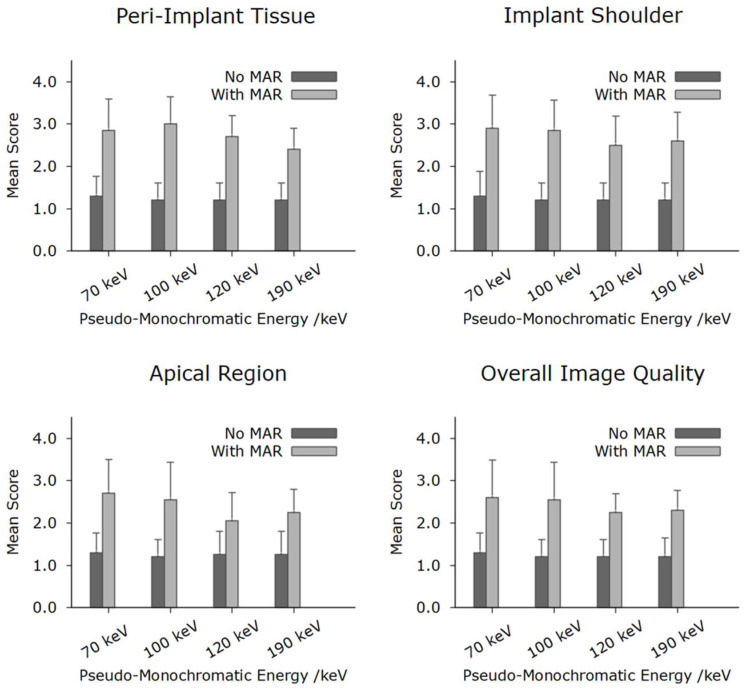
Results of qualitative analysis of the different peri-implant structures. As demonstrated, the non-MAR protocols consistently outperformed the MAR protocols across all evaluated rating categories. Nevertheless, all assessed imaging protocols were ultimately considered diagnostically acceptable.

In total, the reconstructions contained a total of 10 implants. Figure 3 shows a systematic visualization of the different reconstruction protocols in the sagittal, transverse, and axial planes of an implant in region 036. Figure 4 illustrates the image quality scores for the structures of interest, as assessed by the two examiners. In general, the scores for reconstructions are high across all considered structures. Specifically, both evaluators consistently rated the images reconstructed without MAR as predominantly excellent across all anatomical regions assessed. In contrast, the images obtained with MAR were assigned lower quality ratings and were, in some cases, categorized as moderate. Notably, no anatomical structure was rated as poor in any of the image sets. A more detailed evaluation highlighted a consistent limitation in the MAR-processed images: the bone-to-implant interface was often not reliably assessable. Specifically, a hyperdense rim was frequently observed surrounding the implant, presumed to be a byproduct of the MAR algorithm. This rim compromised the evaluation of the peri-implant bone and was not present in reconstructions without MAR. In the non-MAR images, the adjacent bone structures appeared clearly delineated and free of such artifacts (Figure 3), enabling confident assessment. Despite these limitations, the overall image quality in both reconstruction types was sufficient to evaluate all relevant zones. The peri-implant bone, the apical region adjacent to the implant, and the bone at the implant shoulder were well visualized in most cases. Notably, a trend was observed in the MAR-enhanced reconstructions, whereby image clarity and evaluability improved with increasing monochromatic energy. However, this trend was not evident in the non-MAR images, which demonstrated stable image quality across all reconstructed energy levels. The inter-reader reproducibility was excellent for all structures (Table 2). Higher intraclass correlation coefficients (ICCs) were observed for the images without MAR, indicating a more consistent and reliable assessment of image quality and evaluability in these datasets. Overall, the ICC values were very high, with all values exceeding 0.75, which reflects excellent interobserver agreement. Lower ICC values observed in the reconstructions using MAR are consistent with the overall reduced and less reliable image assessment in these datasets, reflecting the increased uncertainty and ambiguity in evaluating image quality and anatomical structures. The ICC values increased with rising monochromatic energy levels in the images with MAR, suggesting that higher monochromatic energies allow for more consistent and reliable assessments, thereby improving the confidence and certainty of image interpretation under MAR conditions.

## 4. Discussion and Conclusions

For the first time, this study examined the imaging quality of peri-implant tissues using a clinically approved PCCT with in vivo clinical data. Our hypothesis was that PCCT provides high image quality for peri-implant structures in vivo. Furthermore, we hypothesized that the application of metal artifact reduction (MAR) algorithms negatively impacts the immediate peri-implant region by reducing image clarity and differentiation.

Both hypotheses were confirmed in this study. Both examiners attested that PCCT delivered excellent results for the visualization of implants in all reconstructions with MAR. This is shown by the excellent median rating scores for all structures assessed accompanied by high ICC values (>0.75). This is consistent with the findings of existing in vitro studies and confirms their promising results. A recent study on implant planning in porcine jaws demonstrated that PCCT fulfilled the clinical precision requirements for implant planning and provided a better image quality as well as reduced artifacts compared with CBCT [9].

The second hypothesis, that MAR reconstructions are inferior to reconstructions without dedicated MAR, was also clearly confirmed. Although these reconstructions still demonstrated moderate to good evaluability, overall image quality was noticeably reduced, and the assessment of peri-implant bone structures was notably poorer. This can be attributed to the nature of MAR algorithms, which attempt to reduce artifacts but may compromise image detail, in particular, in the vicinity of metals. Moreover, adjusting the monochromatic energy level provided little improvement as reflected by the scores of the examiners. These findings are consistent with a previous study investigating the effect of MAR algorithms in CT and CBCT imaging [15]. A recently published study demonstrated that the application of MAR can reduce artifacts caused by dental implants in the surrounding tissues [16]. However, this study does not contradict the findings reported herein, as it focused on different anatomical regions. In the present investigation, the assessment was limited to structures in immediate proximity to the implant. In these areas, the use of MAR resulted in an ambiguous, somewhat blurred depiction of anatomical details (Figure 1). This region can only be interpreted by clinicians with great caution. Precise statements about the condition of the implant–bone interface cannot be made based on these images. However, a rough assessment of the bone trajectory should still be possible. Still, these images can be used for planning purposes, for example, for placing an additional implant adjacent to an existing one, provided they were acquired within a reasonable time frame relative to the implant insertion date. In contrast, reconstructions without MAR provided markedly superior visualization, which is also reflected in the quantitative results. Apart from the implants described here, MAR-corrected images may also be inferior to non-MAR images in other dental diagnostic scenarios, for example, when assessing periodontal structures near metallic restorations as well as in cases of secondary caries adjacent to metallic restorations.

This study is the first to demonstrate in clinical subjects that PCCT is suitable for visualizing peri-implant structures under real-world clinical conditions. While the protocols analyzed in this study were associated with a higher radiation dose compared to established imaging methods in dentistry such as CBCT or intraoral films, this was not the primary focus [6]. Rather, the study emphasizes the opportunistic use of already-acquired data to avoid additional radiation exposure. This principle was successfully demonstrated and opens a new dimension in dental imaging. In the past, conventional clinical CT scans failed to reliably visualize fine dental structures due to their limited spatial resolution [5]. With PCCT, this is now achievable. It will, of course, initially be difficult to translate this idea into clinical reality, since both radiologists and dentists diagnosing this image must be familiar with PCCT dental findings. Training and AI evaluation tools could be extremely helpful here. However, the necessary infrastructure must also be created to enable data exchange between radiologists and dentists. Such structures could initially be tested in smaller use case scenarios, with the aim of making them available to all patients at a later date. In the context of peri-implant structures, for example, this could allow incidental detection of previously unidentified peri-implant defects during spinal examinations, potentially indicating the need for further clinical intervention. One limitation of this study is the absence of a clinical gold standard for validation or a well-defined control group, respectively, i.e., in an ideal case, paired data originating from CBCT and PCCT were available allowing for a direct comparison. However, such data are not available given the risks associated with ionizing radiation. The datasets used herein were obtained from a patient cohort that was not primarily examined in a dental setting. However, previous studies have already demonstrated the accuracy and suitability of PCCT for visualizing osseous structures. Furthermore, the high ICC values observed in this study indicate a clear depiction of anatomical structures, thereby lending strong credibility to the image ratings. Another limitation of the presented study is the available spatial resolution of the system used. Although the in-plane spatial resolution of PCCT in our study approximates that of standard CBCT, the larger nominal slice thickness, i.e., 0.4 mm versus 0.2–0.3 mm in typical CBCT) yields increased voxel volumes, which can introduce partial-volume effects and obscure fine anatomical details. Overlapping reconstructions, e.g., with a slice increment of 0.2 mm as used in this study, help to smooth inter-slice transitions, but do not fully recover the higher longitudinal resolution of thinner-slice acquisitions. Future studies should explore optimized reconstructions or advanced reconstruction algorithms to further enhance the visualization of micro-anatomical and implant-related complexities. Of particular interest is a detector mode referred to as QuantumHD that provides an increased spatial resolution but was not used in the acquisition of the opportunistic data used herein.

Furthermore, given the small sample size used in our study, the findings presented herein must be regarded as preliminary and may reflect random variability. Nonetheless, this investigation provides a foundational framework upon which larger, adequately powered trials can build to confirm and extend our observations.

Given that peri-implantitis, like periodontitis, is associated with a range of systemic conditions, such as diabetes mellitus, cardiovascular diseases, and others, opportunistic imaging could also provide indications of underlying systemic diseases [17]. This approach does not contradict the traditionally dentistry-focused imaging paradigm that remains fully justified and will continue to play a central role. Rather, it should be viewed as a complement and extension of the diagnostic portfolio, offering additional benefits. It has the potential to support more personalized treatment strategies while simultaneously helping to reduce radiation exposure for patients. Furthermore, opportunistic imaging could contribute to more efficient use of healthcare resources by leveraging existing data for broader diagnostic purposes.

In conclusion, this study demonstrates that PCCT paves the way for opportunistic, three-dimensional dental diagnostics within non-dental CT scans, offering the potential for the earlier detection of clinically relevant pathologies of dental implants. This development, in conjunction with advancing AI technologies and the ongoing digital transformation of healthcare, opens up new opportunities for improved patient care, large-scale data analysis, and the discovery of novel medical insights and correlations [18].

## Figures and Tables

**Table 1:** Scan parameters and reconstruction parameters used in the study.

Trajectory	Spiral
CT Dose Index	13.2 mGy (CTDI_32cm_)
Tube Voltage	120 kV
Reconstruction	QIR(3)
Voxel Size	156 µm
Slice Thickness	400 µm
Slice Increment	200 µm
Kernel	Qr64

**Table 2:** The ICC values for the respective categories of the MAR and non-MAR protocols are presented. All values exceed 0.75, indicating excellent inter-rater agreement.

With Metal Artifact Reduction				
	**70 keV**	**100 keV**	**150 keV**	**190 keV**
**Peri-Implant Tissue**	0.914	0.795	1.000	1.000
**Implant Shoulder**	0.846	0.914	1.000	0.916
**Apical Region**	0.851	0.818	0.899	0.842
**Overall Image Quality**	0.815	0.818	0.757	1.000
**Without Metal Artifact Reduction**				
	**70 keV**	**100 keV**	**150 keV**	**190 keV**
**Peri-Implant Tissue**	1.000	1.000	1.000	1.000
**Implant Shoulder**	1.000	1.000	1.000	1.000
**Apical Region**	1.000	1.000	0.842	0.842
**Overall Image Quality**	1.000	1.000	1.000	1.000

## Data Availability

The original contributions presented in this study are included in the article. Further inquiries can be directed to the corresponding author.

## Abbreviations

CNR	contrast-to-noise ratio
CT	computed tomography
CTDI	CT dose index
CBCT	cone beam computed tomography
FOV	field of view
PCCT	photon-counting CT
QIR	Quantum Iterative Reconstruction
MAR	metal artefact reduction
